# Food matrix in the context of muscle and whole-body protein synthesis: a scoping review

**DOI:** 10.1186/s12986-025-00989-y

**Published:** 2025-12-03

**Authors:** Konstantinos Prokopidis, Inga Catharina Brouer, Aaron M. Lett

**Affiliations:** 1 Department of Musculoskeletal Ageing and Science, Institute of Life Course and Medical Sciences, University of Liverpool, Liverpool, UK; 2 Institute and Comprehensive Center for Inflammation Medicine, University of Lübeck, Lübeck, Germany; 3 Department of Metabolism, Digestion and Reproduction, Faculty of Medicine, Imperial College London, London, UK

**Keywords:** Food matrix, Muscle protein synthesis, Skeletal muscle, Food structure

## Abstract

**Background:**

Understanding the impact of food matrix, the combination of nutrients, bioactive compounds, and physical characteristics in a food product, on muscle protein synthesis (MPS), could provide insights on how to use different meals to promote muscle anabolism. The aim of this review was to explore the impact of the food matrix on MPS or whole-body PS.

**Methods:**

PubMed, Scopus, Web of Science, and the Cochrane library were searched from inception until January 2024. A narrative synthesis was employed to synthesize the findings and the quality of the studies was assessed using the Cochrane risk-of-bias 2 tool for randomised trials (RoB 2). The study’s protocol was registered in PROSPERO (International Prospective Register of Systematic Reviews; CRD42024503306).

**Results:**

Only one study was eligible to be included in this review. MPS was 0.035 ± 0.004 %/hr in the minced beef group vs. 0.034 ± 0.003 %/hr in beef steak, postprandially, showing no significant differences between groups. Increased whole-body PS response was observed after the consumption of minced beef compared to beef steak (29 ± 2 mmol phenylalanine/kg vs. 19 ± 3 mmol phenylalanine/kg, respectively; *P* < 0.01). The overall risk of bias was considered low.

**Conclusions:**

Future studies should use food products with identical nutrient composition to evaluate the influence of the food matrix on MPS and whole-body PS, considering that current trials have primarily focused on ensuring similar protein intakes across study arms.

**Supplementary Information:**

The online version contains supplementary material available at 10.1186/s12986-025-00989-y.

## Introduction

Muscle protein synthesis (MPS) is a dynamic process critical for the maintenance, repair, and adaptation of skeletal muscle [3]. MPS incorporates amino acids, the building blocks of proteins, into the muscle tissue, playing a key role in stimulating MPS [13]. Individual factors, including genetics, age, health, and training status, may influence the rate and efficiency of MPS [12, 33].

A large body of evidence has suggested that increased protein intake is an effective strategy to optimise MPS and subsequently supporting skeletal muscle growth and function [23, 34]. Multiple trials have employed different types of protein sources to identify the optimal dose and frequency for maximum anabolism, considering the differing amino acid profiles among protein sources, aiding in the stimulation of MPS [5–7, 14, 18, 21, 24, 25]. In recent years, there has been a growing recognition that the impact of dietary interventions on MPS extends beyond protein dose and source. More specifically, the concept of "food matrix" has been an emerging field, emphasizing the interactive and synergistic effects of nutrients within the complex structure of whole foods.

Whole foods encompass a spectrum of nutrients, including proteins, carbohydrates, fats, vitamins, minerals, and phytochemicals. The food matrix refers to the combination of nutrients, bioactive compounds, and physical characteristics combined within a food product [2]. Understanding how the food matrix influences MPS is becoming an increasing focal point in nutritional research [4], given that humans do not consume isolated nutrients but whole foods. Moreover, the food matrix may influence nutrient bioavailability and digestibility, thus impacting the rate at which amino acids are delivered to the circulation [9]. For example, it has been proposed that slow-digesting proteins, often found in whole foods, may provide a sustained release of amino acids, potentially prolonging the post-prandial anabolic window compared to isolated protein sources such as whey protein, which are rapidly digested [31]. In addition, food processing and cooking methods could alter the food matrix, influencing nutrient bioavailability and structure [27]. Understanding how these processes and modification affect MPS is crucial for optimizing dietary strategies aimed at muscle anabolism, given that the primary mode of food consumption is through whole foods over isolated nutrients. This may be particularly important in individuals with lower protein intake, especially those consuming protein closer to the recommended dietary allowance of 0.8 g/kg/bodyweight, which may be suboptimal for muscle gain optimization. Nevertheless, currently, there are no systematic reviews investigating the impact of the food matrix on MPS or whole-body PS. Therefore, in this review we aim to i) synthesise the current knowledge on the relationship between the food matrix and MPS, offering insights into novel nutritional strategies for optimizing muscle anabolism, and ii) propose methodologies that can be implemented to unravel the potential impact of food matrix on MPS.

## Methods

This scoping review was conducted based on the PRISMA (Preferred Reporting Items for Systematic Reviews and Meta-Analyses) guidelines [17]. The protocol was registered in the PROSPERO (International Prospective Register of Systematic Reviews) database (CRD42024503306).

### Search strategy

Two independent reviewers searched PubMed, Scopus, Web of Science, and the Cochrane library from inception until January 2024. The complete search strategy employed in each database is shown in Table S1. Discrepancies in the literature search process were resolved by a third investigator. The following inclusion criteria were employed: (1) participants irrespective of health status; (2) two or more investigated food products with different food matrix but identical nutrient composition; and (3) available data related to MPS. Studies were excluded if (1) they were not clinical trials; (2) food products did not have an identical nutrient composition; and (3) a full text was not available.

### Data extraction

Two authors extracted data independently, including the name of first author and date of publication, country of origin, study design, participant age, number, and sex, food products of interest, method of MPS assessment, dose, and duration of intervention, and whether dietary intake was assessed at throughout the study. Disagreements between authors were resolved by a third investigator.

### Risk of bias assessment and data synthesis

The quality of the included studies was assessed using version 2 of the Cochrane risk-of-bias 2 tool for randomised trials (RoB 2) and was evaluated by two independent reviewers. Appraisal of risk of bias was performed via the RoB 2 tool included the assessment of the following domains of bias in RCTs: (1) randomisation process, (2) deviations from intended interventions, (3) missing outcome data, (4) measurement of the outcome, and (5) selection of the reported result. In accordance with the RoB 2 tool scoring system, study quality was defined as low risk of bias, some concerns, or high risk of bias. Finally, data from the included studies were qualitatively analysed through narrative synthesis, considering the heterogeneity among food products and the insufficient number of studies.

### Human ethics and consent to participate declarations

Not applicable.

## Results

**Fig. 1:**
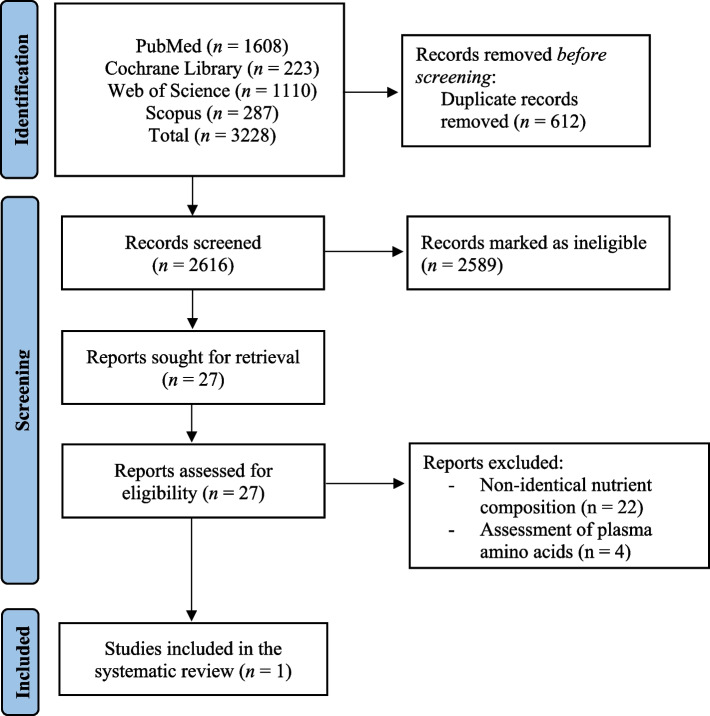
PRISMA flowchart of study selection

The initial literature search yielded 3228 publications. After the exclusion of 612 duplicates, 2616 unique publications were screened. Overall, 2589 publications were marked as ineligible primarily due to comparisons between food groups (intervention vs. comparator), study design, and lack of outcomes of interest. Full-text screening of the remaining five publications resulted in four studies that only assessed plasma amino acid concentration, while 22 studies compared food products with non-identical nutrient composition. Of these, one study was eligible to be included in this review (Fig. 1).

### Characteristics of the included study

The included study assessed MPS and whole-body PS using a steak vs. minced beef in older healthy male participants. The dose was 135 g consisting of 26 g protein, of which, 2 g were leucine. Protein synthetic responses were assessed for 360 minutes following meal consumption. Overall, data was collected from nine participants that consumed steak beef and nine participants in the minced beef group. The concurrent administration of meat ingestion alongside an intravenous infusion of isotopically labeled L-[ring-^2^H_5_]phenylalanine and L-[ring-^2^H_2_]tyrosine was involved. This design aimed to evaluate the kinetics of beef protein digestion and absorption, alongside the assessments of MPS and whole-body PS. The study did not involve an exercise component and none of the subjects had a history of participating in any regular exercise program. A comprehensive description of the included study is presented in Table 1.

**Table 1:** Study and participant characteristics of the included study in the systematic review

Study, year	Country	Study design	Health status	Total	Intervention		Comparator		Treatment and dose	Processing method	Reported outcomes	Assessment Tool	MPS (Duration)
				*n* (M/F)	*n* (M/F)	Age (SD)	*n* (M/F)	Age (SD)					
Pennings, 2013 [19]	Netherlands	Crossover RCT	Healthy	9 (9/0)	9 (9/0)	Total: 74 (2)	9 (9/0)	Total: 74 (2)	Minced beef vs. Steak beef (135 g)	Stored at -18 °C Meals (thawed overnight in a refrigerator at 4 °C) were grilled until the inner temperature reached 65 °C	Mean (+SEM) plasma L-[1-13C]Phe (A) and L-[ring-2H5]Phe (B) enrichments Mean (+SEM) whole-body Phe kinetics Mean (+SEM) whole-body protein metabolism	L-[ring-2 H5]Phe enrichment Protein synthesis = total Rd 2 PHE to TYR conversion PHE net balance = protein synthesis 2 Endo Ra	360 min

*Abbreviations*: *RCT* Randomized controlled trial, *Phe* Phenylalanine, *Rd* Total rate of disappearance of Phe, *TYR* Tyrosine, *Ra* Phe rate of appearance

### Risk of bias assessment

**Fig. 2:**
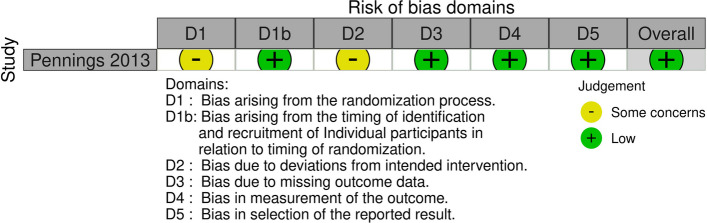
Risk of bias assessment of the included studies

Considering that this was not a blinded study, the included study had some concerns considering the lack of information on how allocation concealment was performed, leading to bias arising from the randomisation process and bias due to deviations from intended intervention. However, the overall risk was considered low. A traffic light plot is presented in Fig. 2.

### Food matrix and muscle and whole-body protein synthesis

Basal mixed muscle protein fractional synthetic rate (FSR) was identical in both the minced and steak groups with 0.035 ± 0.005 %/hr. Postprandially, FSR was 0.035 ± 0.004 %/hr in the minced group and 0.034 ± 0.003 %/hr in the steak group, displaying no significant differences between groups.

No significant differences in whole-body protein synthesis among the studied products was found (*P* > 0.05). However, when examining whole-body protein oxidation, expressed as the area under the curve (AUC) of the Phenylalanine-to-Tyrosine conversion rate, a notable increase was observed after minced beef consumption compared to basal values (*P* < 0.01). Lastly, the assessment of whole-body protein balance indicated a more positive balance after the consumption of minced beef compared to beef steak (29 ± 2 mmol phenylalanine/kg compared with 19 ± 3 mmol phenylalanine/kg, respectively; *P* < 0.01).

## Discussion

In this scoping review, we found only one study that investigated the impact of food matrix on muscle and whole-body protein synthesis. Considering the lack of available studies on the topic, reliable conclusions may not be established. Most studies fail to incorporate study arms aiding in the exploration of the effects of the food matrix on protein synthesis, given that the food products used did not have identical nutrient composition.

In the study by [19] minced beef demonstrated a more positive net protein balance compared to beef steak consumption (*P* < 0.01) [19]. Albeit no differences were found pertinent to plasma glucose, insulin, and leucine levels, this finding may be attributed to the more rapid protein absorption and digestion after ingestion of minced beef vs. beef steak. This was reflected by an increased concentration of exogenous phenylalanine after minced beef vs. beef steak consumption (61 ± 3% vs. 49 ± 3%, respectively) during the postprandial period (6 hours). Considering that MPS remained identical between groups, it is thought that these changes may be explained, in part, due to differences in AA oxidation rates, splanchnic retention of AA from other tissues (i.e., liver, gut), and/or whole-body protein breakdown [11, 26] (Table 2). More rapidly digested proteins may be oxidized at a higher rate, which could reduce the amount of AA available for making new proteins [28], however, research is inconsistent [29]. In addition, similar to our findings, a previous study comparing 30 g of intact milk protein vs. ingestion of a free AA mixture, demonstrated a larger rise in postprandial plasma AA levels, a greater net protein gain, and significantly higher incorporation of dietary phenylalanine into mixed muscle protein [32]. However, there were no observed differences in MPS responses between treatments, likely because the large doses of protein or AA may have already been sufficient to maximize MPS stimulation in healthy young individuals. The mean protein intake from beef consumption in the study by [19] was ~26 g including ~2 g of leucine, which could be sufficient to stimulate MPS sufficiently even in older adults to the extend that the effect of the food matrix may had been negligible. This could also be supported by the lack of difference in plasma leucine levels between groups. Thus, the enhanced net protein balance with minced beef could reflect reductions in whole-body protein breakdown or differences in AA metabolism, rather than changes in MPS, highlighting the food matrix’s role in modulating whole-body protein turnover in older adults [11, 26]. Moreover, considering that the subjects of this trial were older adults, for which, chewing efficiency may be compromised with more solid foods, a greater chewing capacity may have improved meat protein utilisation [22].

**Table 2:** Studies exploring the impact of food matrix on plasma amino acid concentration

Study, year	Country	Study design	Health status	Total	Intervention		Comparator		Treatment and dose	Processing method	Reported outcomes (plasma)	Assessment Tool	Outcome measurement (Duration)
				*n* (M/F)	*n* (M/F)	Age (SD)	*n* (M/F)	Age (SD)					
Agergaard 2021 [1]	Denmark	Crossover RCT	Healthy	6 (3/3)	6 (3/3)	Total: 69 (5)	6 (3/3)	Total: 69 (5)	Steak beef vs. minced beef - 0.55 g protein/kg LBM, served with a mixed meal	The meat used for hydrolysate was minced and mixed up in water and under constant stirring heated to 60 °C. Enzymes (0.1% of meat weight of both the endoprotease Protamex® and the exopeptidase Flavourzyme®, Novozymes, Bagsvaerd, Denmark) were added and the solution was heated under constant stirring: 60 °C for 1 h and subsequently 90 °C for 15 min. The slurry was drained and the pellet (mainly connective tissue proteins) was discarded. The watery hydrolysate was portioned and stored at -40 °C until usage.	- AA - Phe - EAA	Plasma [2H5] phenylalanine enrichment	300 min
Fuchs 2022 [8]	Netherlands	RCT	Healthy	45 (45/0)	15 (15/0)	24 (2)	15 (15/0)	24 (2)	Raw vs. boiled eggs - 5 eggs: 30 g protein	The (hard) boiled eggs were steamed for ∼13 min with 65 mL water in an egg steamer. Participants were allowed to drink 300 mL of water with their eggs.	- AA - NEAA - EAA - BCAA - Leucine	Plasma L-[ring-13C6]-phenylalanine enrichment	300 min
Horstman 2021 [10]	Netherlands	Crossover RCT	Healthy	10 (5/5)	10 (5/5)	66.7 (44.3)	10 (5/5)	66.7 (44.3)	An appropriate amount of 8 different dairy products to ensure 25 g of protein intake (i.e., low or high-fat ultra-high temperature-treated vs. low or high-fat pasteurised milk	To standardise the volume for all products, water was added to a total of 700 mL of volume ingested. For the milk samples, the water was mixed with the milk.	- AA - EAA - Leucine	Liquid chromatography-mass spectrometry (LC-MS) on a EZ:faast AAA-MS column (Phenomex) using a water-methanol gradient	300 min
van Lieshout 2023 [30]	Netherlands	Crossover RCT	Healthy	6 (0/6)	6 (0/6)	Total: 25 (2)	6 (0/6)	Total: 25 (2)	Milk protein concentrate in solid form (protein bar) vs. in liquid form (protein drink) (20 g)	The protein drink was prepared by dissolving 57.1 g of powder in 419 ml of water. Likewise, the protein bar weighed 57.1 g and was consumed together with 419 ml of water.	- AA - EAA - Leucine	Ultra-performance liquid chromatography mass spectrometry (ACQUITY UPLC H-Class with QDa; Waters)	240 min

*Abbreviations*: *RCT* Randomized controlled trial, *LBM* Lean body mass, *AA* Amino acids, Phe Phenylalanine, *EAA* Essential amino acids, *NEAA* Nonessential amino acids, *BCAA* Branched-chain amino acids

A previous study that also compared minced vs. steak beef found greater levels of total AA and EAA following steak at 6 hours postprandially, however, they had different fat composition albeit they were part of an identical macronutrient meal [1]. In addition, following raw vs. boiled egg consumption, boiled eggs elicited significantly greater total AA and EAA concentrations vs. raw eggs [8]. More recently, [30] who compared milk protein concentrate in solid form (protein bar) vs. in liquid form (protein drink) found no changes in any AA concentrations [30], while similar results were shown pasteurised vs. ultra-high temperature heated milk [10]. Based on the aforementioned studies, chewing capacity and its response to whole-body and/or muscle protein utilisation may be food product- as well as cooking processing-specific. Subsequent clinical trials are essential to assess the clinical significance of food texture and effective mastication in preserving muscle mass among the older population.

Furthermore, while current evidence primarily focuses on animal-based proteins such as beef, eggs, and milk, it remains to be seen how the food matrix affects plant-based protein sources, which have been gaining popularity in recent years. Future research should address this gap to better understand the implications for muscle protein synthesis across diverse dietary patterns.

### Strengths, limitations, and future directions

This is the first study that attempts to evaluate the potential impact of food matrix on protein synthesis. However, considering that the majority of trials have utilized food products with similar protein intakes, rather than overall nutrient intakes, the reliability and accuracy of any conclusions based on solely one study could not be established. Furthermore, a systematic review was not able to be performed due to the lack of trials in the field. In addition, MPS represents a mechanistic surrogate rather than a hard clinical endpoint. As such, future studies should consider including more meaningful outcomes such as changes in lean soft tissue, muscle strength, and physical performance to evaluate the long-term relevance of food matrix effects. Moreover, assessing MPS at rest and post-exercise would provide further information into how exercise, particularly resistance training, may interact with the food matrix. In this context, it is worth highlighting that the relevance of food matrix may be more applicable in populations consuming suboptimal amounts of protein. Previous research has indicated minimal benefits of protein diets beyond 1.6 g/kg/bodyweight to support muscle mass and function [15, 16, 20]; therefore, the food matrix may have negligible impact when daily protein intake is already adequate. Trials controlling for dietary intake and experimenting with different protein doses deemed suboptimal would shed further insights into the translational effects of the food matrix on muscle health outside MPS responses.

## Conclusion

This scoping review aimed to explore the impact of food matrix on MPS, for which, only one study met the inclusion criteria. Future studies should incorporate food products with identical nutrient composition in order to assess the influence food matrix may have on MPS, considering that current trials have primarily taken into account only similar protein intakes among study arms. Additionally, future research should move beyond short-term mechanistic outcomes and include clinically relevant endpoints such as LBM and strength, while also exploring the potential moderating role of exercise.

## Supplementary Information

Supplementary Material 1.

## Data Availability

No datasets were generated or analysed during the current study.

## References

[1] Agergaard J, Hansen ET, van Hall G, Holm L. Postprandial amino acid availability after intake of intact or hydrolyzed meat protein in a mixed meal in healthy elderly subjects: a randomized, single blind crossover trial. Amino Acids. 2021;53:951–9. 10.1007/s00726-021-03000-. 33991254 10.1007/s00726-021-03000-z

[2] Aguilera JM. The food matrix: implications in processing, nutrition and health. Crit Rev Food Sci Nutr. 2019;59:3612–29. 30040431 10.1080/10408398.2018.1502743

[3] Atherton P, Smith K. Muscle protein synthesis in response to nutrition and exercise. J Physiol. 2012;590:1049–57. 22289911 10.1113/jphysiol.2011.225003 PMC3381813

[4] Burd NA, Beals JW, Martinez IG, Salvador AF, Skinner SK. Food-first approach to enhance the regulation of post-exercise skeletal muscle protein synthesis and remodeling. Sports Med. 2019;49:59–68. 30671904 10.1007/s40279-018-1009-y PMC6445816

[5] Burd NA, Gorissen SH, van Vliet S, Snijders T, van Loon LJ. Differences in postprandial protein handling after beef compared with milk ingestion during postexercise recovery: a randomized controlled trial. Am J Clin Nutr. 2015;102:828–36. 26354539 10.3945/ajcn.114.103184

[6] Burd NA, Yang Y, Moore DR, Tang JE, Tarnopolsky MA, Phillips SM. Greater stimulation of myofibrillar protein synthesis with ingestion of whey protein isolate v. micellar casein at rest and after resistance exercise in elderly men. Br J Nutr. 2012;108:958–62. 22289570 10.1017/S0007114511006271

[7] Dideriksen K, Reitelseder S, Petersen S, Hjort M, Helmark I, Kjaer M, Holm L. Stimulation of muscle protein synthesis by whey and caseinate ingestion after resistance exercise in elderly individuals. Scand J Med Sci Sports. 2011;21:e372–83. 21535185 10.1111/j.1600-0838.2011.01318.x

[8] Fuchs CJ, Hermans WJ, Smeets JS, Senden JM, van Kranenburg J, Gorissen SH, Burd NA, Verdijk LB, van Loon LJ. Raw eggs to support postexercise recovery in healthy young men: did rocky get it right or wrong? J Nutr. 2022;152:2376–86. 10.1093/jn/nxac174. 36774104 10.1093/jn/nxac174 PMC9644172

[9] Gaudichon C, Calvez J. Determinants of amino acid bioavailability from ingested protein in relation to gut health. Curr Opin Clin Nutr Metab Care. 2021;24:55. 33093304 10.1097/MCO.0000000000000708 PMC7752214

[10] Horstman AMH, Ganzevles RA, Kudla U, Kardinaal AFM, van den Borne JJGC, Huppertz T. Postprandial blood amino acid concentrations in older adults after consumption of dairy products: the role of the dairy matrix. Int Dairy J. 2021;113:104890.

[11] Jonker R, Engelen MPKJ, Deutz NEP. Role of specific dietary amino acids in clinical conditions. Bri J Nutr. 2012;108:S139–48. PMID: 23107525. PMCID: PMC4734127. 10.1017/S0007114512002358 PMC4734127 23107525

[12] McKendry J, Shad BJ, Smeuninx B, Oikawa SY, Wallis G, Greig C, Phillips SM, Breen L. Comparable rates of integrated myofibrillar protein synthesis between endurance-trained master athletes and untrained older individuals. Front Physiol. 2019;10:1084. 31543824 10.3389/fphys.2019.01084 PMC6728413

[13] Mitchell WK, Wilkinson DJ, Phillips BE, Lund JN, Smith K, Atherton PJ. Human skeletal muscle protein metabolism responses to amino acid nutrition. Adv Nutr. 2016;7:828S-838S. 27422520 10.3945/an.115.011650 PMC4942869

[14] Morgan PT, Harris DO, Marshall RN, Quinlan JI, Edwards SJ, Allen SL, Breen L. Protein source and quality for skeletal muscle anabolism in young and older adults: a systematic review and meta-analysis. J Nutr. 2021;151:1901–20. 33851213 10.1093/jn/nxab055 PMC8245874

[15] Morton RW, Murphy KT, Mckellar SR, Schoenfeld BJ, Henselmans M, Helms E, Aragon AA, Devries MC, Banfield L, Krieger JW, Phillips SM. A systematic review, meta-analysis and meta-regression of the effect of protein supplementation on resistance training-induced gains in muscle mass and strength in healthy adults. Br J Sports Med. 2018;52(6):376–84. 10.1136/bjsports-2017-097608. 28698222 10.1136/bjsports-2017-097608 PMC5867436

[16] Nunes EA, Colenso-Semple L, Mckellar SR, Yau T, Ali MU, Fitzpatrick-Lewis D, Sherifali D, Gaudichon C, Tomé D, Atherton PJ, Camprubi Robles M, Naranjo-Modad S, Braun M, Landi F, Phillips SM. Systematic review and meta-analysis of protein intake to support muscle mass and function in healthy adults. J Cachexia Sarcopenia Muscle. 2022;13:795–810. 10.1002/jcsm.12922. PMID: 35187864. PMCID: PMC8978023. 35187864 10.1002/jcsm.12922 PMC8978023

[17] Page MJ, Mckenzie JE, Bossuyt PM, Boutron I, Hoffmann TC, Mulrow CD, Shamseer L, Tetzlaff JM, Akl EA, Brennan SE. The PRISMA 2020 statement: an updated guideline for reporting systematic reviews. Int J Surg. 2021;88:105906. 33789826 10.1016/j.ijsu.2021.105906

[18] Pennings B, Boirie Y, Senden JM, Gijsen AP, Kuipers H, van Loon LJ. Whey protein stimulates postprandial muscle protein accretion more effectively than do casein and casein hydrolysate in older men. Am J Clin Nutr. 2011;93:997–1005. 21367943 10.3945/ajcn.110.008102

[19] Pennings B, Groen BBL, van Dijk J-W, de Lange A, Kiskini A, Kuklinski M, Senden JMG, van Loon LJC. Minced beef is more rapidly digested and absorbed than beef steak, resulting in greater postprandial protein retention in older men. Am J Clin Nutr. 2013;98(1):121–8. 10.3945/ajcn.112.051201. 23636241 10.3945/ajcn.112.051201

[20] Phillips SM, Chevalier S, Leidy HJ. Protein “requirements” beyond the RDA: implications for optimizing health. Appl Physiol Nutr Metab. 2016;41(5):565–72. 10.1139/apnm-2015-0550. 26960445 10.1139/apnm-2015-0550

[21] Reitelseder S, Agergaard J, Doessing S, Helmark IC, Lund P, Kristensen NB, Frystyk J, Flyvbjerg A, Schjerling P, van Hall G. Whey and casein labeled with L-[1-13C] leucine and muscle protein synthesis: effect of resistance exercise and protein ingestion. American Journal of Physiology-Endocrinology and Metabolism. 2011;300:E231–42. 21045172 10.1152/ajpendo.00513.2010

[22] Rémond D, Machebeuf M, Yven C, Buffière C, Mioche L, Mosoni L, Patureau Mirand P. Postprandial whole-body protein metabolism after a meat meal is influenced by chewing efficiency in elderly subjects. Am J Clin Nutr. 2007;85:1286–92. 10.1093/ajcn/85.5.1286. 17490964 10.1093/ajcn/85.5.1286

[23] Stokes T, Hector AJ, Morton RW, McGlory C, Phillips SM. Recent perspectives regarding the role of dietary protein for the promotion of muscle hypertrophy with resistance exercise training. Nutrients. 2018;10:180. 29414855 10.3390/nu10020180 PMC5852756

[24] Tang JE, Moore DR, Kujbida GW, Tarnopolsky MA, Phillips SM. Ingestion of whey hydrolysate, casein, or soy protein isolate: effects on mixed muscle protein synthesis at rest and following resistance exercise in young men. J Appl Physiol. 2009. 10.1152/japplphysiol.00076.2009. 19589961 10.1152/japplphysiol.00076.2009

[25] Tipton KD, Elliott TA, Cree MG, Wolf SE, Sanford AP, Wolfe RR. Ingestion of casein and whey proteins result in muscle anabolism after resistance exercise. Med Sci Sports Exerc. 2004;36:2073–81. 15570142 10.1249/01.mss.0000147582.99810.c5

[26] Tome D. Efficiency of free amino acids in supporting muscle protein synthesis. J Nutr. 2022;152:3–4. PMID: 35021214. 35021214 10.1093/jn/nxab370

[27] Toydemir G, Subasi BG, Hall RD, Beekwilder J, Boyacioglu D, Capanoglu E. Effect of food processing on antioxidants, their bioavailability and potential relevance to human health. Food Chemistry: X. 2022;14:100334. 35712535 10.1016/j.fochx.2022.100334 PMC9194584

[28] Trommelen J, Betz MW, van Loon LJC. The muscle protein synthetic response to meal ingestion following resistance-type exercise. Sports Med. 2019;49:185–97. PMID: 30659499. 30659499 10.1007/s40279-019-01053-5

[29] Trommelen J, van Lieshout GA, Nyakayiru J, Holwerda AM, Smeets JS, Hendriks FK, van Loon LJ. The anabolic response to protein ingestion during recovery from exercise has no upper limit in magnitude and duration in vivo in humans. Cell Rep Med. 2023;4(12). 10.1016/j.xcrm.2023.101324 PMC10772463 38118410

[30] van Lieshout GAA, Trommelen J, Nyakayiru J, van Kranenburg J, Senden JM, Verdijk LB, van Loon LJC. The postprandial plasma amino acid response does not differ following the ingestion of a solid versus a liquid milk protein product in healthy adult females. International Journal of Sport Nutrition and Exercise Metabolism. 2023;33:247–54. 10.1123/ijsnem.2023-0038. 37348850 10.1123/ijsnem.2023-0038

[31] van Vliet S, Beals JW, Martinez IG, Skinner SK, Burd NA. Achieving optimal post-exercise muscle protein remodeling in physically active adults through whole food consumption. Nutrients. 2018;10:224. 29462924 10.3390/nu10020224 PMC5852800

[32] Weijzen ME, Van Gassel RJ, Kouw IW, Trommelen J, Gorissen SH, Van Kranenburg J, Van Loon LJ. Ingestion of free amino acids compared with an equivalent amount of intact protein results in more rapid amino acid absorption and greater postprandial plasma amino acid availability without affecting muscle protein synthesis rates in young adults in a double-blind randomized trial. J Nutr. 2022;152(1):59-67. 10.1093/jn/nxab305 PMC8754581 34642762

[33] Wilkinson DJ, Piasecki M, Atherton PJ. The age-related loss of skeletal muscle mass and function: measurement and physiology of muscle fibre atrophy and muscle fibre loss in humans. Ageing Res Rev. 2018;47:123–32. 30048806 10.1016/j.arr.2018.07.005 PMC6202460

[34] Witard OC, Wardle SL, Macnaughton LS, Hodgson AB, Tipton KD. Protein considerations for optimising skeletal muscle mass in healthy young and older adults. Nutrients. 2016;8:181. 27023595 10.3390/nu8040181 PMC4848650

