# Supplementary material for: Food matrix in the context of muscle and whole-body protein synthesis: a scoping review
Source: Nutr Metab (Lond). 2025 Dec 3;22:151. doi: 10.1186/s12986-025-00989-y (PMC12676753; doi:10.1186/s12986-025-00989-y)
Supplement: Supplementary file 1 — Supplementary Material 1. [file 12986_2025_989_MOESM1_ESM.docx]

**Table S1.**Full search strategy protocol based on title, abstract, and keywords in the literature search.

| **Database** | **Search terms** |
| --- | --- |
|  |  |
| PubMed | (“food structure” OR “food matrix” OR “whole food*” OR chicken OR minced OR steak OR beef OR cheese OR  milk OR kiwi OR potato OR fish OR salmon OR reindeer OR egg OR peas OR cricket OR bean OR soy OR tofu OR  quinoa OR cottage cheese OR dairy OR fung* OR vegetable OR animal)  AND (“muscle protein synthesis” OR “fractional synthetic rate*” OR “whole-body protein synthesis”) |
| Cochrane Library | (“food structure” OR “food matrix” OR “whole food*” OR chicken OR minced OR steak OR beef OR cheese OR  milk OR kiwi OR potato OR fish OR salmon OR reindeer OR egg OR peas OR cricket OR bean OR soy OR tofu OR  quinoa OR cottage cheese OR dairy OR fung* OR vegetable OR animal)  AND (“muscle protein synthesis” OR “fractional synthetic rate*” OR “whole-body protein synthesis”) |
| Web of Science | (“food structure” OR “food matrix” OR “whole food*” OR chicken OR minced OR steak OR beef OR cheese OR  milk OR kiwi OR potato OR fish OR salmon OR reindeer OR egg OR peas OR cricket OR bean OR soy OR tofu OR  quinoa OR cottage cheese OR dairy OR fung* OR vegetable OR animal)  AND (“muscle protein synthesis” OR “fractional synthetic rate*” OR “whole-body protein synthesis”) |
| Scopus | (“food structure” OR “food matrix” OR “whole food*” OR chicken OR minced OR steak OR beef OR cheese OR  milk OR kiwi OR potato OR fish OR salmon OR reindeer OR egg OR peas OR cricket OR bean OR soy OR tofu OR  quinoa OR cottage cheese OR dairy OR fung* OR vegetable OR animal)  AND (“muscle protein synthesis” OR “fractional synthetic rate*” OR “whole-body protein synthesis”) |
